# The Effect of Maternal Overweight and Obesity Pre-Pregnancy and During Childhood in the Development of Obesity in Children and Adolescents: A Systematic Literature Review

**DOI:** 10.3390/nu14235125

**Published:** 2022-12-02

**Authors:** Adriana Mannino, Katerina Sarapis, George Moschonis

**Affiliations:** Department of Food, Nutrition and Dietetics, School of Allied Health, Human Services & Sport, La Trobe University, Melbourne, VIC 3086, Australia

**Keywords:** pediatric obesity, maternal obesity, pre-pregnancy BMI, obesogenic environment

## Abstract

Maternal overweight/obesity has been associated with an increased risk of obesity in childhood. We investigated the effect of maternal overweight/obesity during pre-pregnancy and whether it is a stronger predictor of child obesity, compared to maternal overweight/obesity during childhood. Prospective or retrospective cohort studies published in English, reporting on obese children and adolescents (2–18 years), with overweight/obese mothers in either pre-pregnancy or during childhood were included. A search was conducted from 2012 to April 2022 in MEDLINE, Web of Science, CINAHL, and EMBASE, followed by screening, data extraction, quality assessment and narrative synthesis. Eleven eligible studies (9 prospective and 2 retrospective cohort studies; total sample, *n* = 27,505) were identified. Eight studies examined maternal overweight/obesity in pre-conception, presenting consistent positive associations with childhood obesity, three reported positive associations between childhood obesity and maternal overweight/obesity during childhood, and one presented positive associations between both maternal exposures. The narrative synthesis failed to identify which maternal exposure is the strongest predictor of childhood obesity, with studies reporting significant associations between maternal overweight/obesity and child obesity in both time points. Intervention programs aiming to reduce childhood obesity should focus on supporting women of childbearing age with weight management from preconception and throughout their life-course.

## 1. Introduction

Obesity represents a global public health problem that has tripled in prevalence since 1975, with an estimated 13% of the world’s adult population considered as obese. Similarly, the prevalence of overweight and obesity has risen dramatically among children and adolescents with an estimated 74 million boys and 50 million girls aged 5–19 years being affected by obesity worldwide [1].

The development of obesity is an interplay of complex exposures of biological, behavioural, and psychosocial origins [2,3,4,5]. Obesity pathogenesis includes two factors; when energy intake exceeds energy expenditure, where a sustained positive energy balance results, and a second factor, a key barrier to treatment of obesity, where the ‘set point’ of body weight is adjusted to an increased value [6]. Diet greatly impacts the risk of obesity due to high caloric intake. However, there are also environmental factors including sedentary lifestyles, environmental exposures, as well as developmental factors such as genetic and epigenetic factors which predispose risk to obesity [6].

Overweight and obesity have long lasting physical, mental and emotional adverse effects on children as they move into adolescence and then to adulthood [7], along with an increase in risk for chronic diseases, orthopaedic impacts and cardiometabolic disorders [8,9]. Furthermore, extensive evidence suggests strong links between childhood obesity and maternal obesity at different stages of the maternal life-course [10], with the majority of the data focusing on the presence of maternal obesity at pre-conception [5], and during childhood [11].

A recent systematic literature review and meta-analysis found that children born to mothers that were obese before conception had a 264% increase in the odds of obesity [5]. Several mechanistic pathways occurring early in life have been proposed in order to interpret the associations observed between maternal pre-pregnancy obesity with the occurrence of obesity and related comorbidities in offspring. In this context, the Thrifty Phenotype hypothesis, which was firstly proposed by Hales and Barker [12] 30 years ago, describes the relationship between periconceptional and early growth risk factors with long-term health, along with permanent changes in glucose-insulin metabolism, leading to the development of type 2 diabetes and metabolic syndrome. Since this was postulated, there is vast evidence demonstrating the influence of prenatal and childhood exposures of adiposity and long-term health status in later life [13,14].

The association between maternal weight status post-pregnancy and childhood obesity has also been investigated in previous studies [10,11,15,16,17,18], which reported increased risk of obesity in children of overweight/obese mothers. High maternal body mass index (BMI) is thought to affect child obesity not only through shared genetic predispositions but also through environmental factors such as an obesogenic home [19,20]. More specifically, parents and especially mothers have an important role in shaping eating behaviours and excess weight in their offspring [21] with the food landscape at home and maternal food preferences greatly influencing children’s food choices, therefore increasing the risk for the development of childhood obesity [22]. In addition to unhealthy dietary patterns, low levels of physical activity and increased sedentary lifestyles have been also described as vital behavioural determinants of the ‘obesogenic environment’ that is linked to childhood obesity [13]. In this regard, children of overweight or obese mothers are more likely to be exposed to an environment that supports and sustains a positive energy balance, thus increasing the likelihood for childhood obesity [11].

Although several studies have investigated maternal obesity as a risk factor for childhood obesity, the vast majority have examined the effect of maternal overweight and/or obesity at singular time points, such as the pre-pregnancy [23,24], during pregnancy [25], post-natal [11], and during childhood [26,27], while there is limited evidence on the combined effect of these exposures at different time points [18,28,29]. To our knowledge, there are currently no known systematic reviews synthesising the relevant evidence, as a means to define which one of the two exposures deserves more attention in the design of childhood obesity prevention programs. This could provide further understanding of the most appropriate timing of preventative strategies and interventions to reduce the incidence of childhood obesity. Hence, the aim of this systematic literature review (SLR), was to identify and synthesise the evidence from observational cohort studies that focus on the effect of maternal pre-conception overweight/obesity and maternal overweight/obesity after the child’s birth on the development of childhood obesity, thus also demonstrating the exposure with the stronger effect.

## 2. Materials and Methods

This systematic review was conducted according to the PRISMA 2020 statement guidelines [30]. The review protocol was registered and published by PROSPERO on 12 May 2022 (Registration number: CRD42022325667); and can be accessed via: https://www.crd.york.ac.uk/prospero/display_record.php?RecordID=325667. Ethics was approved by the La Trobe University Human Research Ethics Committee (Ethics Approval Number: HEC22199).

### 2.1. Eligibility Criteria

The selection of appropriate inclusion and exclusion criteria for this SLR was guided by PECO(S) [30]. In this context, the inclusion criteria were set out as follows; The population (P), examined children and adolescents aged 2–18 years of age. The exposure (E) included mothers with overweight or obesity in either the pre-pregnancy period or during the child’s life-course/during childhood. The comparison (C) included mothers in the same period with normal weight BMI cut-offs. The primary outcomes (O) were childhood obesity as defined by the World Health Organisation (WHO) or the Centres for Disease Control (CDC) growth standards. The secondary outcomes included child and adolescent anthropometric measurements, such as BMI, BMI-for-Age Z-scores, waist circumference (WC), body fat % (%BF), and fat mass index (FMI). Finally, the study design (S), sought observational cohort studies (prospective and retrospective studies); along with the high-level of evidence cohort studies provide, this review aimed to determine causality, i.e., the association between the exposure (maternal weight status/obesity) and the outcome (childhood obesity).

Studies were eligible for inclusion if they were human participant studies, written in English and published in peer reviewed journals within 10 years (>2012). This date range was determined by an initial search of relevant SLRs, which identified relevant cohort studies published from 2012 onwards and to reflect current population data [14,31]. No minimum duration of follow up was prescribed. Studies which contained data from both infants or adults (<2 years or >18 years), along with data on children and adolescents (2–18 years) were included if the child and adolescent data was reported separately. Studies were excluded if they presented results for infants (<2 years) or adults >18 years), or if they used non-human participants. Studies were also excluded where the exposure was parental obesity, with mother and father’s weight status combined. Additionally, studies in languages other than English were also excluded along with articles without a full text.

### 2.2. Search Strategy and Information Sources

A comprehensive systematic literature search was conducted using PubMed/MEDLINE, Web of Science, CINAHL (Cumulative Index of Nursing and Allied Health Literature) via EBSCO, and EMBASE/Ovid for studies published in English, with identical limits for publication dates, language and age applied. Hand searching/citation mining of reference lists was undertaken to identify additional studies not found in the database searches, including protocol papers for included studies. The first date searched was 13 April 2022 and the last search was 15 April 2022. Subject headings and key words were combined using truncations and Boolean Operators for each database can be found in Tables S2–S5. Key words related to the study population, exposure, outcome, and study design categories were combined with ‘OR’; while the key words within each category group were combined with ‘AND’. The BMJ Best Practice Evidence Based Toolkit was used to design search filters to retrieve specific cohort study records [32].

### 2.3. Study Selection

**Figure 1:**
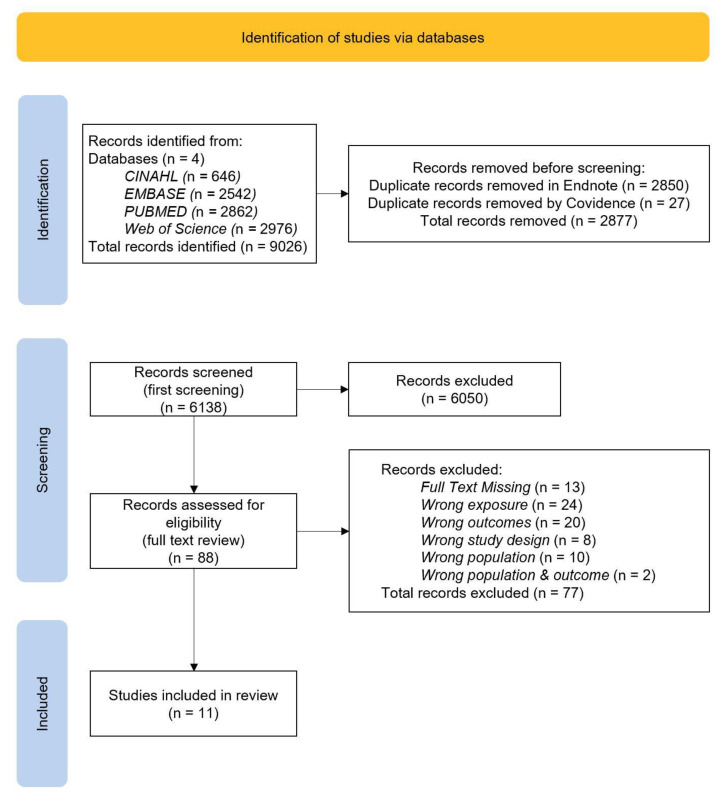
Flowchart of the search; Prisma Diagram.

A systematic search of the aforementioned databases was conducted by A.M. as per the protocol. Results were exported to EndNote (Version 20) [33], where duplicate records were reviewed and removed systematically based on the ‘Bramer method of de-duplication’ [34]. Records were exported from Endnote, and subsequently imported to Covidence, a systematic review management software package, where remaining duplicates were automatically removed [35]. Titles and abstracts were then screened by A.M., with irrelevant references removed based on ineligibility. The full-text review was conducted for the resulting studies by three reviewers, A.M., K.S. and G.M. using the inclusion and exclusion criteria. For records with missing full text, a search of general search engines was undertaken. Where a full text was unable to be located, those studies were excluded. All conflicts were resolved through discussion and final consensus was obtained. The final number of included records was decided by all reviewers. Reasons for exclusion were recorded and reported in Figure 1.

### 2.4. Data Collection Process and Data Items

A data extraction template was designed in Covidence Extraction 2.0. outlining variables under the category of study details, participant characteristics, exposure/outcomes, and results, which were determined to be the most critical domains for studies and to assist with a comprehensive rich summary for data synthesis and critical appraisal.

To ensure a rigorous data extraction process and calibration, two reviewers, A.M. and K.S. independently reviewed the first five studies in Covidence. Data items collected included publication information (title, journal, year of publication, author names and affiliations, funding sources, conflict of interest), study details (study design, location and setting, aim, statistical analysis method), study participant characteristics (sample size, child age, sex, maternal exposure period), Exposure and Outcomes (primary Outcome(s), Secondary Outcome(s)), results (statistical power/significance), conclusions (key findings, strengths, limitations). The results of the initial extraction were discussed and compared to ensure alignment in the research team. The remaining studies were extracted independently by A.M. based on the consensus and expectations formed in the initial extraction. Once the extraction was complete, the results were exported to an Excel Spreadsheet for data synthesis.

### 2.5. Risk of Bias and Quality Assessment in Individual Studies

The risk of bias assessment, at an outcome and study level, was first reviewed by two reviewers, A.M. and K.S., who independently reviewed the first five studies in Covidence. The balance of the risk of bias assessment was completed by A.M. Risk of bias was assessed using the Quality Criteria Checklist for Primary Research, developed by the Academy of Nutrition and Dietetics (formerly known as the ADA Checklist) [36]. This checklist assesses accuracy, relevancy, validity, and generalisability of the included studies; providing an overview of the selection and comparability of the study groups, withdrawals, blinding, exposures, outcomes, statistical analysis and conflict of interest, the checklist is also applicable to level 2 and 3 evidence [37]. Studies which resulted in the majority of responses being “yes” received a positive result, indicative of a low risk of bias. A negative result occurred when there were six or more responses of “no”, which indicated a high risk of bias. Missing or unclear data on conflict-of-interest statements, funding and affiliations resulted in an increase in the risk of bias, based on assumptions of a lack of transparency by study authors.

### 2.6. Summary Measures

To report the association between maternal obesity and childhood obesity, we extracted relevant association coefficients, i.e., odds ratio (OR), relative risk (RR) and their 95% Confidence Intervals (95% CIs). In addition, the associations between maternal obesity and continuous measures of weight status in children (i.e., BMI, WC, and BF), were reported via beta coefficients and their 95% CIs, means and standard deviations (SDs). These measures allowed for ease of interpretation of the results and the most appropriate for the study design included in this review. The review describes characteristics for participant and location information, data collection and analysis methods. The extracted data identified key information from individual studies. The outcome of interest for this review was childhood obesity (as measured by high BMI and/or high WC levels), for children with mothers who are obese or overweight at various timepoints in their maternal life-course, with a special emphasis in the period before the child’s conception (pre-pregnancy) and at the child’s current age (i.e., while the child grows up).

### 2.7. Data Synthesis

To describe the direction and strength of the associations between exposures (maternal overweight/obesity/maternal weight status) and the examined outcomes, the review presents a narrative synthesis completed by A.M. of association coefficients reported by the selected studies, along with their key features. This review used the SWiM (Synthesis without meta-analysis) in systematic reviews reporting guideline to conduct the synthesis, to promote transparent reporting and as an extension to the PRISMA 2020 checklist [38].

### 2.8. Meta-Bias/Risk of Bias across Studies

To control for meta-bias in individual studies and assess for publication, reporting or selection bias, where methodologies are unclear, missing or not identified, we reviewed the primary/original study or protocol papers cited in the study. Where study protocols were unavailable, the outcomes reported in the methodology and result sections of the published report were compared and reviewed. If study protocols were available, the outcomes reported in the protocol and published report were compared by reviewers.

## 3. Results

### 3.1. Study Selection

Figure 1 shows the results retrieved from the four databases included in this review, with 9026 studies identified in total. Of these, 2850 were removed in Endnote using a de-duplication removal process. Additionally, Covidence detected and removed 27 more records. This resulted in 6138 records which proceeded to title and abstract screening. The initial screening found 6050 irrelevant studies. From 88 studies which were included in the full text review, 77 were excluded, as assessed with the inclusion and exclusion criteria. Studies were excluded for having a full text missing or the wrong population, exposure outcome, or study design. Finally, 11 studies were found to be eligible for inclusion in this review.

### 3.2. Study Characteristics

Of the 11 studies included in this systematic review, nine comprised prospective cohort studies, while the remaining two were retrospective cohort studies. We categorised child and adolescent age groups based on the CDC stages of child development [39]. Of the prospective cohort studies, four studies were conducted among pre-school children (aged 3–5) [40,41,42,43]; two studies were conducted among children of middle childhood age (6–11 years) [44,45]; one study examined both the toddler (2–3 years) and the pre-school age groups (aged 3–6) [46]; one study was conducted across both the pre-school (aged 3–5) and middle childhood age groups (6–11 years) [47]; two studies were conducted across the middle childhood (6–11 years) and young teens group (12–14 years) [48,49]; and finally, one study covered toddlers (2–3 years) to young teens collectively (12–14 years) [50]. The studies range across six countries, two in Brazil, one from China, two from Greece, one from Japan, one from Turkey, and four from the United States. Summarised study characteristics are available in Table 1.

#### 3.2.1. Children’s Outcome Measures

Within the individual studies, primary and secondary outcomes were reported, and those that produced statistically significant results were examined. From these, nine studies explored relevant primary outcomes [40,42,44,45,46,49] and two explored secondary outcomes [43,48]. The outcomes included childhood obesity a categorical variable, as defined by WHO and CDC, and child body composition measures (i.e., BMI, BMI-for-Age Z-Score, WC, FMI and %BF). Two groups were used to categorise the outcomes measured, named “categorical outcome measures” and “continuous outcome measures”. These have been presented in Table 1. Five studies explored childhood obesity as a categorical variable [41,43,47,49,50], and six studies explored child anthropometric and body composition measures as continuous variables [40,42,44,45,46,48].

#### 3.2.2. Maternal Exposures

For the exposures, eight studies examined maternal pre-pregnancy overweight or obesity [40,41,42,44,45,46,47,49], and three studies examined maternal overweight or obesity during childhood [43,47,48,50], while one study [47] examined both exposures. The effect measures used to examine the associations between maternal exposures and children’s outcome measures included beta coefficients (β), RRs, ORs, mean differences, means and correlation coefficients. Statistics of precision for these effect measures (i.e., Standard Errors of the Mean, 95% CIs) were also presented. Statistical significance was set at *p* < 0.05 for all studies.

### 3.3. Results of Individual Studies

#### 3.3.1. Studies on Child Body Composition (Continuous Variables)

Six studies reported positive associations between maternal weight status and childhood continuous anthropometric and body composition variables (i.e., BMI, WC, FMI, %BF) [40,42,44,45,46,48]. Of these four studies examined child BMI-for-Age Z-Scores [40,44,46,48]; two studies examined WC [42,44]; and two studies examined %BF [45,46]. Childhood BMI was examined in only one prospective cohort study [42]. Studies examining associations between maternal pre-pregnancy overweight, or obesity and Child BMI-for-Age Z Score also reported positive associations [40,44,46]. Studies examining associations between maternal pre-pregnancy overweight/obesity with children’s continuous outcome measures of weight status (i.e., BMI, WC) reported beta coefficients (β) [40,44,46]. Ehrenthal et al. showed a significant positive association between pre-pregnancy maternal obesity (obese, BMI > 30 kg/m^2^ and very obese, BMI > 40 kg/m^2^) and child BMI-for-Age Z-scores (β 0.497, 95% CI 0.382, 0.611 and β 0.755, 95% CI 0.636, 0.874, respectively) [40].

Another study reported similar results, using BMI-Z scores, with higher mean values observed for children with overweight/obese mothers compared to children with normal weight mothers (*p* < 0.05) [46]; however the study by Dias et al. reported non-significant associations between pre-pregnancy maternal obesity and child BMI-for-Age Z-Scores (β 0.83, 95% CI 0.64, 1.01) [44]. The study by Xu and colleagues investigated the association between maternal overweight or obesity as the child grows up and child’s weight status [48]. Results demonstrated that children with an obese mother had an increasing annual rate of 0.41% (95% CI 0.01%, 0.84%) for %BF, 0.76 cm (95% CI 0.28 cm, 1.25 cm) for WC, and 0.29 kg/m^2^ (95% CI 0.13 kg/m^2^, 0.45 kg/m^2^) for BMI, compared to children with normal weight mothers.

Studies examining associations between maternal pre-pregnancy overweight/obesity and children’s WC also reported significant associations [42,44]. Daraki and colleagues reported associations between maternal pre-pregnancy overweight/obesity with central obesity in children (i.e., WC > 90th percentile), showing that the risk of central obesity in children was almost twice as high in those whose mothers were overweight or obese before pregnancy (RR 1.97; 95% CI 1.11, 3.49) [42]. Two other studies examined associations between maternal pre-pregnancy overweight and obesity with children’s FMI. In one of these studies, mean FMI was higher in children with obese and overweight mothers before pregnancy, compared to children with normal-weight mothers pre-pregnancy (5.3 ± 3.0 vs. 4.5 ± 2.4 vs. 4.0 ± 2.0, respectively, *p* < 0.001) [45]. The second study also reported significant positive associations between children’s FMI and maternal pre-pregnancy overweight (β 3.83, 95% CI 2.56, 5.11) and obesity (β 6.84, 95% CI 4.82, 8.87) [44]. In addition, two studies examined the associations between maternal pre-pregnancy BMI and child’s %BF. In this context, Andres et al. reported a greater increase in %BF in boys with obese mothers at the age of 2 years, compared to boys born with mothers who were normal-weight and overweight prior to conception (4.8% vs. 0.9%, respectively) [46].

Castillo and colleagues also showed a direct association between maternal pre-pregnancy BMI and child’s %BF [45]. In specific, the mean %BF was higher in children with overweight and obese mothers before pregnancy, compared to those with normal-weight mothers (24.7 ± 8.4 vs. 26.8 ± 9.5 vs. 23.0 ± 7.5) *p* < 0.001), respectively). Additionally. the study by Kjaer et al. found maternal pre-pregnancy obesity to be significantly associated with childhood obesity at the age of 9 in obese children vs. non-obese (32% vs. 11%, respectively; *p* = 0.002) [47]. We also found some gender differences across the selected studies, with three studies reporting significant associations of childhood obesity and increased anthropometric and body composition measures (BMI, FMI and WC) in girls only, when mothers were obese before the child’s conception [42,44,46].

#### 3.3.2. Studies on Childhood Obesity Status (Categorical Variables)

Five studies explored the association between maternal weight status and childhood obesity status as a categorical variable [41,43,47,49,50]. Of these, two studies examined the effect of maternal pre-pregnancy weight status [41,49]; two other studies the effect of maternal weight status during childhood [43,50]; and one study the effect of both exposures [47]. All studies reported significant positive associations between maternal weight status and childhood obesity. More specifically, two prospective cohort studies showed increased risk for obesity in children born by mothers that were overweight/obese before pregnancy [41,49]. The study by Zafar Janjua et al. found that overweight and obese mothers were 2.30 times (RR 2.30; 95% CI: 1.29, 4.11; *p* = 0.005) and 2.53 times (RR 2.53; 95% CI 1.49–4.31; *p* < 0.001) more likely to have obese children compared to normal-weight mothers [41].

Dhana et al. also similarly found that for overweight and obese mothers, children were more likely to be obese (RR 2.83; 95% CI 2.35, 3.42; and RR 4.15; 95% CI 3.36, 5.13) [49]. Moreover, the study by Kjaer et al. found at 3, 4, and 5 years post-partum, maternal obesity was associated with child obesity at 9 years (52% vs. 29%, *p* = 0.01; 59% vs. 27%, *p* = 0.001; and (48% vs. 28%, *p* = 0.03, respectively) [47]. Regarding the effect of maternal overweight/obesity on children’s weight status during childhood, two retrospective cohort studies found increased likelihoods of childhood obesity when mothers were also obese, [43,50]. Specifically, in the study by Vehapoglu, Goknar, Turel, Torun and Ozgurhan [50], obese mothers had a 3.91 times higher likelihood of having children who were obese (95% CI 2.02, 5.93). Similarly in the study by Kato et al. (2014), mothers who were obese were more likely to have a daughter who was obese (OR 3.11; 95% CI: 1.54, 6.27; *p* < 0.001), but this association was not found in sons (OR 1.01; 95% CI: 0.31, 3.35; *p* = 0.99).

### 3.4. Risk of Bias within Studies

Risk of bias was assessed for each study using the Academy of Nutrition and Dietetics Quality Criteria Checklist for Primary Research, to determine possible bias. From the 11 studies included in this systematic review, seven studies received a positive overall score, representative of a low risk of bias [40,41,42,45,46,49,50]. Four studies were found to have a neutral overall score, representative of a medium risk of bias [43,44,47,48]. Selection bias was identified in studies that did not describe or provide detailed inclusion and/or exclusion criteria [44,46,48]. Low generalisability was another source of bias in the studies identified by this review, due to small sample sizes [47], convenient sample selection, and sampling procedures that did not render a representative sample [40,41,43,47,49,50]. Recall bias was found in studies with self-reported maternal pre-pregnancy BMI [40,41,45,46,48,50]. Sponsorship bias was also identified for studies that did not provide a clear statement of declaring there was ‘no conflict of interest’ [40,42,44,49].

Confounding bias was identified for studies which may have not controlled for relevant and/or appropriate confounders [40,43,44,47,49]. Ehrenthal et al. used electronic medical record data primarily for their analysis presenting possible systematic and random errors [40], and Dhana et al. did not describe the exposure in detail [49]. Reporting bias was identified for those studies that did not discuss withdrawal rates [40,42,46,49]; and attrition bias was found in studies with a withdrawal rate of >20% [41,47]. Across all studies included in this systematic review there were no studies which discussed blinding of outcomes as it was not applicable for this study design and therefore was assessed as not applicable. Sponsorship bias was identified where studies did not provide a clear statement of declaring there was ‘no conflict of interest’ [40,42,44,49].

The sources of bias, along with overall scores for the risk of bias and quality are presented in Table 1. Additionally, responses to individual relevancy and validity questions from the Quality Criteria Checklist for Primary Research checklist are recorded for individual studies in Table 2. Regarding meta-bias or risk of bias across the studies, this systematic review did not detect publication or selective reporting bias.

## 4. Discussion

To our knowledge, this is the first systematic review of prospective and retrospective cohort studies to examine the effects of the two maternal exposures, i.e., maternal pre-pregnancy overweight/obesity and maternal overweight/obesity during childhood, on childhood obesity. The studies included in this review used both continuous and categorical outcome variables, as well as a range of effect measures that made the assessment of which exposure might have the strongest effect of the examined outcome not feasible. Regarding both exposures for childhood obesity, amongst the 11 cohort studies investigated in this systematic review, eight out of the 11 which examined maternal weight status before conception, showed positive associations with childhood obesity, and three out of the 11 studies reported positive associations between childhood obesity and maternal weight status during childhood. Furthermore, one study provided results demonstrating associations between both exposures, but did not provide data that could allow comparisons and the assessment of which exposure is stronger [47].

In line with the findings of the current review [40,44,46], previous studies [5,14] have shown consistently that maternal pre-pregnancy overweight/obesity status was positively associated with childhood obesity, further highlighting the importance of preconception maternal weight status in offspring’s long-term health [31]. A recent study by Choi and colleagues found paternal pre-pregnancy obesity was associated with a 3.11 times increased risk of childhood obesity in children aged 3–5 years old. Higher maternal pre-pregnancy BMI levels have been also reported to exert a strong effect on child and adolescent obesity, describing this stage as, when the exposure pertaining to genetic and epigenetic and developmental programing factors occurs in critical embryonic stages, has long lasting implications for offspring metabolic regulation [18]. The specific mechanism involved is related to the increased inflammation, insulin resistance and high blood glucose levels in overweight and obese mothers before, and as such, during pregnancy, which result in exposing the growing fetus to a hostile, over-nutritional intrauterine environment [10,52,53]. An additional early life factor to consider would be the dietary patterns of mothers who are overweight/obese in the preconception stage, which may also influence risk of childhood adiposity and obesity. Moreover, a birth cohort study found adherence to a Mediterranean diet, considered to be a healthy dietary pattern, was associated with lower child WC [54].

Our study found eight studies showing associations between maternal pre-pregnancy overweight and obesity and childhood obesity. Furthermore, five studies [40,42,44,45,46] found similar associations with childhood obesity measured by high anthropometric measurements and body composition. This was also found by Heslehurst and colleagues, where the findings of their meta-analysis saw increasing maternal BMI associated with continuous child BMI and z-score outcomes [5].

This review found four studies which reported positive associations between maternal obesity during childhood and childhood obesity. During childhood there are a number of factors which could contribute to the development of obesity, mainly related to the fact that mothers moderate children’s energy-balance related behaviors [13]. Energy balance which is reflected in physical activity levels and dietary intake are the most direct downstream determinants of childhood obesity. The obesogenic environment that is usually created and sustained by overweight and obese parents, influences children’s eating patterns and behaviors, primarily affected by role modelling of parents throughout childhood; and although genetic predisposition influences a child’s obesity risk and metabolism, genetic risks can be amplified or weakened by gene-environment interactions [55,56]. Moreover, there is a complex cycle within the obesogenic environment, obesity during childhood, and pre-conception obesity, where there is potential exposure of mothers to obesogenic factors, in turn influencing not only the in utero environment, but the obesogenic environment for their offspring [5].

Interestingly, this review found studies which showed that in the presence of maternal overweight and obesity, girls were at higher risk of obesity and higher body composition measures compared to boys [42,43,44]. This finding also aligns to the literature, which suggests varying pathways to obesity in each one of the two sexes, such as differences in pubertal timing and energy requirements [55].

While most of the studies included in this review examined both exposures, few studies provided long-term follow up evaluation in childhood and/or adolescence, with the exception of three studies [48,49,50]. Moreover, most of the included studies reported results for short-term follow up periods, after following up offspring until their pre-school age. Pre-adolescence is a critical life stage with an increasing number of potential exposures, which differ from childhood, and shape lifestyle behaviors and food choices [8]. The age of obesity onset is an important factor to consider for children and adolescents where there may be significant impacts on the child’s long-term health. Obesity in the pre-adolescent stage (approximately 7 years of age) has been associated with an increased risk of type 2 diabetes in midlife and overall mortality from cardiovascular diseases; this risk is significantly increased if BMI increases between the age of 7 and the onset of puberty [57,58,59]. Moreover, the risk of cardiovascular disease is significantly increased when obesity is present in early life and persists into adolescence [57,60,61].

### 4.1. Strengths and Limitations

This systematic review has many strengths; the rigorous search strategy was developed with the assistance of a specialist librarian. This review was conducted according to the requirements of the PRISMA Statement [30]. To minimise subjectivity and increase rigor in the review, the full text reviews were conducted by three reviewers, while the use of a systematic review management software (Covidence) was another means to reinforce objectivity. Another strength of this review is the use of studies with observational cohort designs, as this is considered level 2 and 3 evidence according to the classification provided by the National Health and Medical Research Council [37]. Moreover, cohort studies have the potential to provide the strongest scientific evidence due to the ability to provide evidence that is indicative of causality between exposures and outcomes. Additionally cohort studies can examine multiple exposures simultaneously [62]. Another strength is the inclusion mainly of prospective cohort studies (i.e., 9 out of 11) in the review, instead of retrospective ones. Strengths of individual studies included the use of important covariates, study design, use of validated measures, and length of follow up.

This systematic review also had limitations, as it was challenging to interpret the evidence found due to the varying outcome and effect measures used across the 11 studies. Regarding the exposures, this systematic review included studies examining maternal obesity and did not set a criterion for BMI thresholds, although most studies used international cut-offs for overweight and obesity. Obesity is universally defined by BMI which also has inherent limitations when applied at an individual level, where it does not distinguish between muscle and fat mass and may overestimate adiposity. In children, the definition of obesity based on a wide range of country-specific or international growth standards, is another limitation which reflects the variability in the way the studies included in this review have defined the study outcome. The variability in the different body composition measures reported for the cohorts of children participating in the selected studies also represents another limitation, since it constrains comparability across studies.

The presence of publication bias in the present study, could not be avoided with the consideration of unpublished data or studies which not included in review due to the search date. Furthermore, due to the high level of heterogeneity of the selected studies, a meta-analysis could not be conducted. Lastly, another limitation of the present review is the use of prospective and retrospective cohort studies, where observational studies do not allow for causal inferences. Limitations of individual studies included low generalisability, sample size, attrition bias, and recall bias.

### 4.2. Implications and Recommendations

The prevention of childhood obesity requires a multifactorial approach with further prospective studies needed to determine which maternal exposure could be the stronger predictor for childhood obesity. As discussed, the findings of this systematic review align to previous reviews and observational studies which show significant associations for each exposure with the development of childhood obesity. Overall, this review has shown the strong relationship with maternal weight status as an exposure. Although previous studies have shown maternal pre-pregnancy obesity to be a stronger predictor of childhood obesity [5,14,18], when compared to other exposures, we are yet to determine whether it is a stronger predictor when compared to maternal obesity during childhood.

Obesity in children and adolescence can affect long-term health and intervention studies have shown that reduction in body weight prior to puberty can reduce the risk of cardiometabolic diseases later in life [57,63].

Overall to influence a change in the intergenerational obesity seen globally, public health policy should look to target obesity prevention and intervention at the pre-conception stage, and for future pregnancies, and tailor public health messages to all women of childbearing age [5,64,65]; providing messaging to them on the importance of pre-conception weight management, as well as in keeping a healthier body weight and dietary pattern throughout their life course.

## 5. Conclusions

In summary, there is currently insufficient evidence to conclude which maternal exposure is a stronger effect for the development of childhood obesity. However, this review has confirmed the multifactorial etiology of childhood obesity, indicating that maternal overweight and obesity has an important role in the development of childhood obesity, regardless of its occurrence (i.e., before the child’s conception or during childhood). While there is clear indication of the effect of maternal overweight and obesity in both the pre-pregnancy period and during childhood, further prospective cohort studies are required to assess the exact effect of both exposures. The results of the current review show that obesity prevention programs should start as early in life as possible and should continue throughout childhood, focusing among others, in helping women retain a healthier body weight throughout their life course.

## Figures and Tables

**Table 1:** Characteristics and data synthesis of included studies (*n* = 11).

Author, Year, Study Type, Location	Sample		Maternal Exposure Timepoint		Child Outcomes	Methodology		Results	Quality
	*n*	Age (Y)	Life-Cycle Time Point	Weight Measures	Weight Measures	Statistical Method	Confounding Factors	Size of Effect	Risk of Bias [36] and Limitations
**Continuous Outcome Measures**									
Xu et al. (2019). Prospective; China [48]	2066	6–14	During Childhood	OW/OB	BMI-Z (>90th%) %BF BMI(kg/m^2^) WC (cm)	PROC MIXED	Paternal and maternal education, parent’s obesity status and BMI	Child BMI-Z (>90th%) and M-WS Mean difference (95% CI) **DC-OW** 0.19 (0.06, 0.32), *p* ≤ 0.001 **DC-OB** 0.33 (0.09, 0.57), *p* ≤ 0.001 Child %BF (%) and M-WS **DC-OW** 0.38 (95% CI 0.16, 0.61), *p* = 0.001 **DC-OB** 0.41 (95% CI 0.01, 0.84), *p* = 0.001 Child BMI (kg/m^2^) and M-WS **DC-OW** 0.21 (95% CI 0.12, 0.29), *p* ≤ 0.001 **DC-OB** 0.29 (95% CI 0.13, 0.45), *p* ≤ 0.001 Child WC (cm) and M-WS **DC-OW** 0.57 (95% CI 0.32, 0.83), *p* ≤ 0.001 **DC-OB** 0.76 cm (95% CI 0.28, 1.25), *p* ≤ 0.001	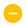 NEUTRAL - Selection Bias - Recall Bias
Ehrenthal et al. (2013). Prospective; United States [40]	3302	4	Pre-pregnancy	OW OB VOB	BMI-Z	Bonferroni Post hoc estimation	Child’s age Maternal height	Child BMI-Z and M-WS β (95% CI) **PP-OW** 0.261 (0.169, 0.355) **PP-OB** 0.497 (0.382, 0.611) **PP-VOB** 0.755 (0.636, 0.874) *p* ≤ 0.001	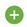 POSITIVE - Recall Bias - Reporting Bias - Low Generalisability - Measurement error - COI not declared
Dias et al. (2021). Prospective; Brazil [44]	3467	10.9 (0.3 SD)	Pre-pregnancy	UW NW OW OB	BMI-Z WC (cm) %FMI	Linear regression	Family income at birth, maternal age, parity, maternal schooling, and maternal smoking during pregnancy.	Child BMI-Z and M-WS β (95% CI) **PP-OW** 0.33, (0.22, 0.45) **PP-OB** 0.83, (0.64, 1.01) Child WC (CM) and M-WS β (95%CI) **PP-OW** 3.21, (2.07, 4.34) **PP-OB** 6.95, (5.17, 8.72) Child %FMI and M-WS β (95% CI) **PP-OW** 3.83, (2.56, 5.11) **PP-OB** 6.84, (4.82, 8.87)	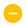 NEUTRAL - Selection Bias
Daraki et al. (2015). Prospective; Greece [42]	879	5	Pre-pregnancy	OW/OB (BMI > 25 kg/m^2^)	BMI WC (cm) WC (%)	Poisson and Linear regression	Child sex, height & TV watching at 4 Y; Maternal age, education, parity, smoking during pregnancy, gestational weight gain, birth weight, breast-feeding duration	Child BMI and M-WS β (95% CI) (model 3) **PP-OW/OB** 0.79 (0.36, 1.06) Child WC % and M-WS RR (95% CI) (model 3) **PP-OW/OB** 1.97 (1.11, 3.49) Child WC (cm) and M-WS β (95% CI) (model 3) **PP-OW/OB** 1.36 (0.55, 2.17)	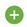 POSITIVE - Reporting Bias - COI not declared
Castillo et al. (2015). Prospective; Brazil [45]	3156	6	Pre-pregnancy	NW OW OB (BMI)	FM (kg) FMI %BF	Linear regression	Family income, Maternal Schooling, skin colour, age, parity, pre-gestational arterial hypertension, pre-gestational diabetes, Pp-BMI	Child FM (KG) and M-WS N, Mean (SD) **PP-NW** 1914, 5.9 (3.2) **PP-OW** 751, 6.9 (4.0) **PP-OB** 352, 8.0 (5.2) *p* ≤ 0.001 Child FMI and M-WS N, Mean (SD) **PP-NW** 1890, 4.0 (2.0) **PP-OW** 747, 4.5 (2.4) **PP-OB** 349, 5.3 (3.0) *p* ≤ 0.001 Child %BF and M-WS N, Mean (SD) **PP-NW** 1914, 23.0 (5.6) **PP-OW** 751, 24.7 (8.4) **PP-OB** 352, 26.8 (9.5) *p* ≤ 0.001	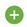 POSITIVE - Recall Bias
Andres et al. (2015). Prospective United States [46]	325	3–6	Pre-pregnancy	NW OW OB	BMI-Z %BF	Linear or restricted cubic splines	Race, gestational age, birth weight, mode of infant feeding	Child BMI-Z (5&6 Y) (Daughter) and M-WS **PP-OW/OB** Higher Scores, compared girls of NW Mothers, *p* ≤ 0.05 Child BMI-Z (3–6 Y) (Son) and M-WS **PP-O** Higher Scores compared to boys of OW or NW Mothers, *p* ≤ 0.05 Child %BF (2–6 Y) (Daughter) and M-WS **PP-OB** Higher %BF by 1.52% from, compared to girls from NW Mothers, *p* ≤ 0.05. Child %BF (2 Y) (Son) and M-WS **PP-OB** Higher %BF by 6 Y, by 4.8%, compared to boys from OW and NW Mothers (0.9%), *p* ≤ 0.05.	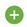 POSITIVE - Recall Bias - Selection Bias - Reporting Bias
**Categorical Outcome Measures**									
Dhana et al. (2018). Prospective; United States [49]	5701	9–14	Pre-pregnancy	OW (25–29.9 kg/m^2^) OB (BMI > 30 kg/m^2^)	WS OB	Multivariable log-binominal regression models with generalised estimating equations	Mothers, age at birth, race/ethnicity, parity, pre-pregnancy alcohol intake, educational attainment of spouse/partner	Child OB and M-WS RR (95%CI) **PP-OW** 2.83 (2.35, 3.42) Child OB and M-WS RR (95%CI) **PP-OB** 4.15 (3.36, 5.13)	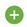 POSITIVE - Reporting Bias - Low Generalisability
Zafar Janjua et al. (2012). Prospective; Greece [41]	740	5	Pre-pregnancy	LW&NW OW OB (BMI)	WS OB (>90th%)	Log binomial regression, Poisson regression with robust variance estimation	Mothers age, race, years of schooling, total number children in family, total number adults in home, employment status of mother, financial assistance, smoking status	Childhood OB and M-WS RR (95%CI) **PP-OW** 2.30 (1.29, 4.11), *p* = 0.005 Childhood OB and M-WS RR (95%CI) **PP-OB** 2.53 (1.49, 4.31), *p* ≤ 0.001; Normal ARR 1.00	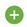 POSITIVE - Low Generalisability - Recall Bias - Increased Attrition Bias
Kjaer et al. (2019). Prospective; United States [47]	201	5–9	Pre-pregnancy & During Childhood	OB BMI	OB	Multivariable logistic regression	Nil	Child OB (9 Y) and M-WS OR (95%CI) **PP-OB** 1.09 (1.00, 1.18), *p* = 0.04 Child OB compared to non-obese (9 Y) and M-WS (4 Y Postpartum) N/total [%]) **DC-OB** 25/48 [52%] vs. 24/82 [29%], *p* = 0.01 Child OB compared to non-obese (9 Y) and M-WS (4 Y Postpartum) N/total [%]) **DC-OB** 24/41 [59%] vs. 20/74 [27%], *p* = 0.03 Child OB compared to non-obese (9 Y) and M-WS (5 Y Postpartum) N/total [%]) **DC-OB** 21/44 [48%] vs. 19/69 [28%], *p* = 0.03 Child OB compared to non-obese (9 Y) and M-WS N/total [%]) **PP-OB** 17/53 [32%] vs. 10/90 [11%], *p* = 0.002	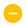 NEUTRAL - Low Generalisability - Low Sample Size - Increased Attrition Bias
Vehapoglu et al. (2017). Retrospective; Turkey [50]	4990	2–14	During Childhood	NW OB (BMI > 30.0 kg/m^2^)	BMI OB	Multiple binary logistic regression	Model 2: Child age, gender, Mode of delivery, Breastfeeding duration, Timing of solid foods initiation Model 3: Maternal education level, smoking during pregnancy.	Child OB and M-WS OR (95%) (Model 2) **DC-OB** 3.91 (2.02–5.93) Child OB and M-WS OR (95%) (Model 3) **DC-OB** 3.84 (1.92–5.88)	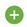 POSITIVE - Low Generalisability
Kato et al. (2014). Retrospective; Japan [43]	2678	5	During childhood	OB (BMI > 25 kg/m^2^)	WS OB (BMI > 90th%)	Chi-square test and Cochran-Armitage test	Nil	Child OB and M-WS (Total) OR (95%CI) **DC-OB** 2.14 (1.19, 3.86), *p* ≤ 0.01; AR = 5.8 Child (Daughter) OB and M-WS OR (95%CI) **DC-OB** 3.11 (1.54, 6.27), *p* ≤ 0.001; AR = 9.8 Child (Son) OB and M-WS OR (95%CI) **DC-OB** 1.01 (0.31, 3.35), *p* = 0.99; AR = 0.1	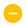 NEUTRAL - Low Generalisability

*n*, Number; Y, Years of Age; OW/OB, Overweight and Obese; P, *p*-Value; M-WS, Maternal Weight Status Categorical); BMI-Z, Body Mass Index-for-Age Z-Score; 90th%, 90th Percentile; 95%CI, 95 % Confidence Interval; cm, centimeter; DC-OW, Maternal overweight during childhood; DC-OB, Maternal obesity during childhood; β, beta coefficient; PP-OW, Maternal pre-pregnancy overweight; PP-OB, Maternal pre-pregnancy obesity; PP-VOB, Maternal pre-pregnancy very obese; SD, standard deviation; %FMI, Fat Mass Index %; BMI, Body Mass Index; WC, Waist Circumference, (centimeters); UW, Underweight (BMI < 18.5 kg/m^2^); NW, Normal weight (BMI > 18.5–24.9 kg/m^2^); OW, Overweight (BMI 25–29.9 kg/m^2^); OB, Obesity (BMI > 30 kg/m^2^) [51]; LW&NW, Low weight and normal weight; FM (Kgs), Fat Mass, kilograms; %BF, Body fat percentage; RR, risk ratio; OR, odds ratio; ARR, absolute relative risk; 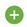, Overall Positive Risk of Bias Score, If most of the answers to the above validity questions are “Yes” (including criteria 2, 3, 6, 7 and at least one additional “Yes”; 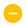, Neutral Risk of Bias Score, If the answers to validity criteria questions 2, 3, 6, and 7 do not indicate that the study is exceptionally strong [36].

**Table 2:** Risk of Bias Assessment [36].

		Zafar Janjua et al. (2012) [41]	Xu et al. (2019) [48]	Vehapoglu et al. (2017) [50]	Kjaer et al. (2019) [47]	Kato et al. (2014) [43]	Ehrenthal et al. (2013) [40]	Dias et al. (2021) [44]	Dhana et al. (2018) [49]	Daraki et al. (2015) [42]	Castillo et al. (2015) [45]	Andres et al. (2015) [46]
	OVERALL RATING	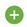	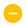	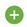	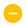	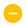	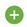	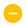	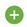	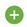	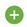	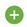
	RELEVANCE QUESTIONS											
1	Would implementing the studied intervention or procedure (if found successful) result in improved outcomes for the patients/clients/population group? (NA for some epidemiological studies)	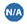	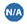	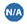	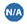	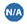	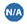	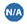	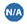	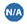	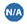	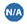
2	Did the authors study an outcome (dependent variable) or topic that the patients/clients/population group would care about?	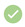	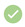	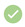	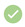	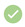	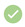	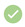	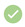	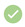	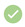	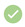
3	Is the focus of the intervention or procedure (independent variable) or topic of study a common issue of concern to dietetics practice?	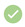	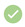	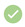	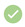	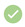	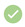	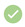	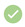	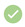	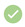	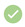
4	Is the intervention or procedure feasible? (NA for some epidemiological studies)	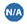	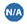	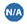	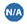	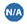	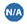	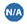	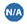	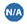	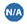	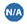
	VALIDITY QUESTIONS											
1	Was the research question clearly stated?	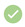	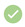	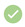	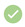	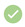	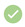	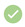	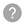	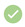	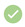	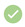
2	Was the selection of study subjects/patients free from bias?	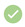	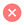	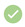	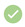	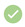	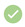	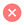	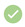	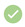	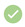	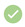
3	Were study groups comparable?	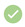	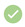	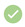	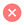	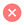	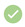	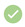	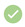	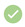	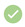	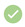
4	Was method of handling withdrawals described?	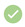	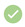	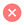	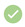	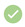	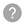	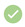	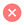	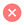	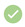	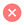
5	Was blinding used to prevent introduction of bias?	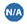	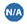	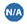	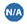	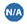	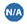	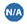	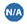	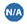	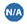	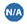
6	Were intervention/therapeutic regimens/exposure factor or procedure and any comparison(s) described in detail? Were intervening factors described?	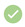	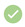	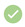	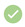	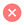	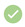	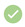	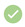	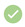	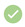	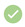
7	Were outcomes clearly defined and the measurements valid and reliable?	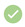	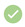	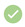	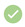	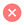	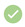	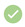	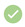	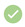	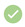	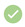
8	Was the statistical analysis appropriate for the study design and type of outcome indicators?	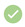	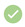	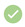	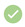	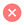	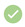	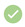	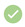	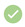	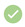	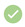
9	Are conclusions supported by results with biases and limitations taken into consideration?	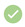	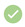	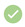	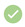	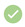	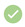	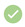	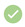	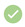	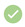	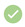
10	Is bias due to study’s funding or sponsorship unlikely?	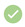	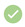	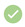	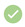	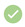	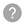	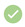	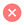	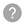	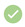	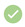

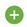
 Overall Positive Risk of Bias Score, If most of the answers to the above validity questions are “Yes” (including criteria 2, 3, 6, 7 and at least one additional “Yes”; 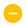 Neutral Risk of Bias Score, If the answers to validity criteria questions 2, 3, 6, and 7 do not indicate that the study is exceptionally strong; 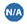 not applicable; 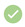 Yes; 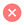 No; 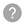 Unclear [36].

## Data Availability

Not applicable.

## Supplementary Materials

The following supporting information can be downloaded at: https://www.mdpi.com/article/10.3390/nu14235125/s1, Table S1: The Quality Criteria Checklist for Primary Research; Table S2: PubMed Search Strategy; Table S3: CINAHL/EBSCO search strategy; Table S4: Web of Science search strategy; Table S5: EMBASE/OVID search strategy. Reference [36] are cited in the Supplementary Materials.
