# Supplementary material for: The Effect of Maternal Overweight and Obesity Pre-Pregnancy and During Childhood in the Development of Obesity in Children and Adolescents: A Systematic Literature Review
Source: Nutrients. 2022 Dec 2;14(23):5125. doi: 10.3390/nu14235125 (PMC9739272; doi:10.3390/nu14235125)
Supplement: Supplementary file 1 [file nutrients-14-05125-s001.zip › nutrients-2024167-supplementary.pdf]

Supplementary Materials

Table S1. The Quality Criteria Checklist for Primary Research.

	**RELEVANCE QUESTION***
1	Would implementing the studied intervention or procedure (if found successful) result in improved outcomes for the patients/clients/population group? (NA for some Epi studies)
2	Did the authors study an outcome (dependent variable) or topic that the patients/clients/population group would care about?
3	Is the focus of the intervention or procedure (independent variable) or topic of study a common issue of concern to dietetics practice?
4	Is the intervention or procedure feasible? (NA for some epidemiological studies)
	**VALIDITY QUESTION***
1	Was the research question clearly stated?
2	Was the selection of study subjects/patients free from bias?
3	Were study groups comparable?
4	Was method of handling withdrawals described?
5	Was blinding used to prevent introduction of bias?
6	Were intervention/therapeutic regimens/exposure factor or procedure and any comparison(s) described in detail? Were intervening factors described?
7	Were outcomes clearly defined and the measurements valid and reliable?
8	Was the statistical analysis appropriate for the study design and type of outcome indicators?
9	Are conclusions supported by results with biases and limitations taken into consideration?
10	Is bias due to study’s funding or sponsorship unlikely?

*Adapted from the Quality Criteria Checklist for Primary Research [36].

Table S2. PubMed Search Strategy.

Search #		Search Statement	Term Type
Population	1	pediatrics OR child OR adolescent OR offspring	MeSH Headings
Exposure	2	maternal obesity OR mothers OR prenatal development OR parent OR Prenatal Exposure Delayed Effects OR "Embryonic and Fetal Development"	MeSH Headings
	3	"Maternal weight status" OR "Maternal overweight" OR Postpartum OR Postnatal OR Prepregnancy OR Preconception OR Pregravid OR "parent* obes*" OR “During Childhood” OR obesogenic	Keywords
		2 OR 3	-
	4	obesity OR overweight OR body composition OR body mass index OR body weight OR waist circumference OR Body-Weight Trajectory OR body mass index	MeSH Headings
	5	"Body-weight trajectory" OR BMI	Keywords
	6	4 OR 5	-
Outcome	7	childhood obesity OR adolescent obesity	MeSH Headings
	8	“Weight for height” OR “BMI for age”	Keywords
	9	8 OR 9	-
Study Design	10	cohort studies OR retrospective studies OR prospective studies OR observational studies	MeSH Headings
	11	3 AND 4 AND 7 AND 10 AND 13<br><br>Limits: (From 2012/1/1 to 2022/4/14, Humans, English, Preschool Child: 2-5 years, Child: 6-12 years, Adolescent: 13-18 years)	-

*Truncation (to expand spellings and multiple endings of a word); PubMed/MEDLINE database coverage: 1965 to present. Last searched 13 April 2022.

Table S3. CINAHL/EBSCO search strategy.

	Search #	Search Statement	Limits
Population	1	Pediatrics+ OR Child+ OR Adolescence+	MeSH Headings
Exposure	2	obesity, maternal+ OR mothers+ OR maternal exposure+ OR fetal development+ OR parents+ OR prenatal exposure delayed effects+ OR postnatal period+	All fields
	3	"pre*gravid" OR maternal overweight OR "pre-conception" OR "pre-pregnancy" OR "during childhood" OR "Maternal weight status" OR "parent* obes*" OR Obe-sogenic	All fields
	4	Obesity+ OR Body Composition+ OR Body Mass Index OR Waist Circumference OR Body Mass Index	MeSH Headings
	5	"overweight" OR "body-weight trajectory" OR BMI	All fields
	6	2 OR 3 OR 4 OR 5	-
Outcome	7	Pediatric Obesity	MeSH Headings
	8	adolescent obesity OR childhood obesity OR BMI for age OR weight for height	All fields
	9	7 OR 8	-
Study De-sign	10	(MH "Epidemiological Research+")<br>(MH "Retrospective Design")<br>(MH "Prospective Studies+")	MeSH Headings
	11	cohort? OR follow up OR longitudinal OR AB retrospective$ OR observational	Abstract
	12	10 OR 11	-
	13	1 AND 6 AND 9 AND 12<br>Limits: (English Language; Published Date: 2012-2022, Human	All fields

+, explode results; *Truncation (to expand spellings and multiple endings of a word); CINAHL/EBSCO database coverage: 1982 to present. Last searched 15 April 2022.

Table S4. Web of Science search strategy.

	Search #	Search Statement	Limits
Population	1	Pediatrics OR Child OR Adolescence	Topic
Exposure	2	maternal obesity OR parent obes* OR maternal overweight OR Pre*pregnancy OR "During Childhood" OR Pre*conception OR obesogenic OR prenatal development	Topic
	3	body composition OR body weight OR waist circumference OR Body-Weight Trajec-tory OR body mass index OR BMI OR "Weight for height" OR "BMI for age"	Topic
	4	2 AND 3	-
Outcome	5	child obesity OR adolescent obesity	Topic
Study Design	6	cohort OR longitudinal OR retrospective OR prospective	Abstract
	7	1 AND 4 AND 5 AND 6<br>Limits: (Publication date: 2012-2022 Languages: English<br>MeSH Headings: Humans)	-

*Truncation (to expand spellings and multiple endings of a word); Web of science database coverage: 1900 to present. Last searched 15 April 2022.

Table S5. EMBASE/OVID search strategy.

Search #		Search Statement	Term Type
Population	1	Pediatrics+ OR child+ OR adolescent+ OR juvenile+	MeSH Headings
	2	p?ediatric* OR Child* Adolesc* OR teen* OR juvenile* OR youth*	Keywords
	3	1 OR 2	-
Exposure	2	maternal obesity+ OR mother+ OR prenatal development+ OR parent+ OR Prenatal Exposure Delayed Effects+ OR "Embryonic and Fetal Development"+ OR obesogenic environment+	MeSH Headings
	3	"Maternal obes*" OR "Maternal weight status" OR "Maternal overweight" OR mother* OR Postpartum OR Post*natal OR Pre*pregnancy OR Pre*gravid OR "parent* obes*" OR 'During Childhood'	Keywords
	4	2 OR 3	-
	5	Obesity+ OR overweight+ OR body composition OR body mass+ OR body weight+ OR waist circumference+ OR Body-Weight Trajectory+ OR body mass index+	MeSH Headings
	6	obes* OR "body composition" OR "body mass index" "waist circumference" OR "Body-weight trajectory" OR BMI	Keywords
	7	5 OR 6	-
Outcome	8	childhood obesity+ OR adolescent obesity+	MeSH Headings
	9	"child* obes*" OR "adolesc* obes*" OR "Weight for height" OR "BMI for age"	Keywords
	10	8 OR 9	-
Study Design	11	Epidemiology OR cohort analysis OR follow up OR longitudinal study OR retrospective study OR prospective study OR observational study	MeSH Headings
	12	epidemiologic OR cohort? OR follow up OR longitudinal OR retrospective$ OR prospective$ OR observational	Keywords *Limit to title and abstract*
	13	11 OR 12	-
	14	3 AND 4 AND 7 AND 10 AND 13 Limits: (Human and English language and yr="2012 -Current")	-

+, explore results; *Truncation (to expand spellings and multiple endings of a word); Embase/Ovid database coverage: 1947 to present. Last searched 14 April 2022.
